# The Impact of First-Trimester Vaginal Microecology on Spontaneous Abortion: A Retrospective Cohort Study

**DOI:** 10.3390/pathogens15090916

**Published:** 2026-08-31

**Authors:** Wenyu Lin, Lihua Chen, Jun Shen, Liying Wang, Qi Chen, Siqi Cao, Liangpu Xu, Na Lin

**Affiliations:** 1 Fujian Provincial Key Laboratory of Prenatal Diagnosis and Birth Defect, Medical Genetic Diagnosis and Therapy Center, Fujian Maternity and Child Health Hospital, College of Clinical Medicine for Obstetrics & Gynecology and Pediatrics, Fujian Medical University, Fuzhou 350001, China; lwyfjmu@outlook.com (W.L.); chenqi.135@outlook.com (Q.C.); 18450075203@163.com (S.C.); 2 Laboratory of Gynecologic Oncology, Fujian Maternity and Child Health Hospital, College of Clinical Medicine for Obstetrics & Gynecology and Pediatrics, Fujian Medical University, Fuzhou 350001, China; chenlh@fjmu.edu.cn (L.C.); wly17200605824@163.com (L.W.); 3 Fujian Key Laboratory of Women and Children’s Critical Diseases Research, Fujian Maternity and Child Health Hospital (Fujian Women and Children’s Hospital), Fuzhou 350001, China; 4 Fujian Clinical Research Center for Gynecological Oncology, Fujian Maternity and Child Health Hospital (Fujian Obstetrics and Gynecology Hospital), Fuzhou 350001, China; 5 Fujian Provincial Cervical Disease Diagnosis and Treatment Health Center, Fujian Maternity and Child Health Hospital, College of Clinical Medicine for Obstetrics & Gynecology and Pediatrics, Fujian Medical University, Fuzhou 350001, China; shenjun559@163.com

**Keywords:** vaginal microecology, abortion, pregnancy complications

## Abstract

**Background:** Spontaneous abortion is a complex multifactorial disease. Recently, vaginal microbiota has become a research hotspot for understanding the etiology of spontaneous abortion. Here, we aimed to explore associations of first-trimester vaginal microecology with adverse pregnancy outcomes. **Methods:** A retrospective cohort study included 786 pregnant women receiving prenatal care at Fujian Maternity and Child Health Hospital between 2023 and 2025. All participants underwent vaginal microecological examinations. Study outcomes included abortion, preterm birth (PTB), prelabor rupture of the membranes (PROM), intrapartum fever, meconium-stained amniotic fluid, etc. The logistic regression analysis was used to estimate odds ratios (ORs) and 95% confidence intervals (CIs) for the associations between vaginal microecological parameters and these outcomes. **Results:** Of 786 participants, 160 (20.4%) had spontaneous abortion, and 626 (79.6%) delivered. The mean vaginal *Lactobacillus* proportion was significantly lower in the spontaneous abortion group (42.5 ± 35.85%) than in the non-abortion group (61.6 ± 26.72%; *p* < 0.001). After age adjustment, vulvovaginal candidiasis (VVC) (OR = 2.17, 95% CI = 1.34–3.52, *p* = 0.001), bacterial vaginosis (BV) (OR = 3.19, 95% CI = 1.89–5.38, *p* < 0.001), and moderate-to-severe aerobic vaginitis (AV) (OR = 2.71, 95% CI = 1.08–6.82, *p* = 0.034) were associated with elevated spontaneous abortion risk. Among enzymatic markers, inflammation-related leukocyte esterase was also associated with higher spontaneous abortion risk (OR = 2.22, 95% CI = 1.39–3.61, *p* < 0.001). In addition, BV was significantly associated with PROM (OR = 2.95, 95% CI = 1.50–5.79, *p* = 0.002). **Conclusion:** Reduced *Lactobacillus* abundance and pathogenic proliferation linked to BV and VVC constitute a risk factor for spontaneous abortion. These findings suggest the potential clinical value of vaginal microecological monitoring in early-trimester pregnancy.

## 1. Introduction

Spontaneous abortion is defined as fetal loss before 28 weeks of gestation. When a woman experiences two or more spontaneous abortions, the condition is commonly referred to as recurrent spontaneous abortion (RSA) [1,2]. The risk of experiencing a spontaneous abortion in a pregnancy is estimated to be 15.3% of all confirmed pregnancies, with a 95% confidence interval ranging from 12.5% to 18.7% [3]. Spontaneous abortion imposes considerable physical and psychological burdens on affected women [4].

Many factors, such as genetic factors, structural uterine abnormalities, hormonal and metabolic dysfunctions, autoimmune disorders, infections, risk factors (age, female body-mass index (BMI), and ethnicity), poor lifestyle choices (smoking, caffeine, alcohol, and work pattern) and environmental risk factors contribute to spontaneous abortion [5]. Despite extensive investigations into these etiological factors, nearly 50% of spontaneous abortion cases remain unexplained, posing a significant challenge for both clinicians and researchers [6,7]. Reproductive tract infections represent the second most common cause of spontaneous abortion following genetic abnormalities, accounting for up to 15% of first-trimester miscarriages [8]. Accumulating evidence suggests that the composition and relative abundance of vaginal microbiota play crucial roles in regulating pregnancy outcomes [9].

The vaginal microbiome is a dynamic ecosystem shaped by a multitude of endogenous and exogenous factors. A *Lactobacillus* spp.-dominant vaginal microbiota is recognized to exert a protective role, whereas vaginal dysbiosis has been associated with spontaneous abortion [10]. Vaginal dysbiosis induces local inflammatory responses, which in turn predispose to vaginosis, sexually transmitted infections (STIs), implantation failure, spontaneous abortion, PTB, and PROM. Although the underlying mechanisms are not fully understood, there is evidence that the makeup of the vaginal microbiota has been linked to a higher risk of spontaneous abortion [11].

With the rapid development of sequencing technologies, a growing number of microorganisms colonizing the female genital tract have been identified and characterized. However, clinically, vaginal microecology [12] is typically evaluated by assessing the morphological and functional parameters of vaginal contents, including pathogens (e.g., *Trichomonas vaginalis*, fungi, and clue cells), vaginal cleanliness, vaginal pH, leukocyte esterase, and sialidase. Therefore, this study aims to explore the relationship between first-trimester vaginal microecology and adverse pregnancy outcomes, which may offer theoretical references for the early identification and timely management of vaginal dysbiosis.

## 2. Materials and Methods

### 2.1. Study Population

**Figure 1:**
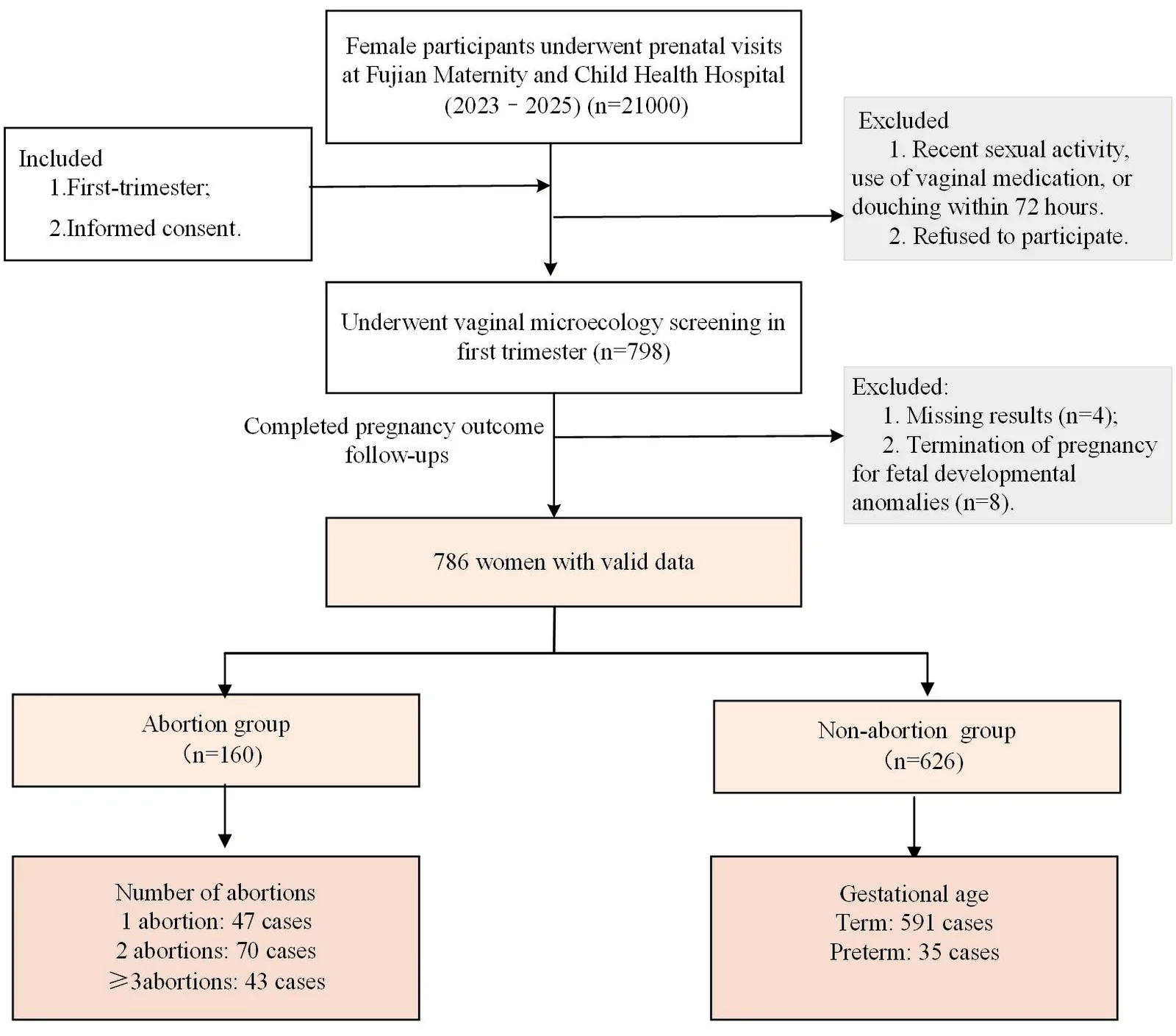
Flowchart. This flowchart depicts the recruitment of pregnant women who underwent prenatal visits at Fujian Maternity and Child Health Hospital. Ultimately, 786 women with valid data were categorized into two groups: the abortion group and the non-abortion pregnancy group.

This retrospective cohort study included female participants attending prenatal visits at Fujian Maternity and Child Health Hospital from 2023 to2025. Eligible participants were in the first trimester of pregnancy and provided informed consent. Exclusion criteria included: (1) recent sexual activity, use of vaginal medication, or douching within 72 h prior to specimen collection; (2) refusal to participate in the study (Figure 1). Eligible individuals underwent vaginal microecology screening (*n* = 798) in first trimester. Additionally, participants with missing follow-up results (*n* = 4) or those who underwent pregnancy termination due to fetal developmental anomalies (*n* = 8) were excluded. Ultimately, a total of 786 women with complete valid data were included in the study and stratified into the spontaneous abortion group (*n* = 160) and the non-abortion pregnancy group (*n* = 626). This study was approved by the Ethics Committee of Fujian Maternity and Child Health Hospital, Affiliated Hospital of Fujian Medical University (Approval No. 2023KY027). The procedures followed were in accordance with the ethical standards of the Declaration of Helsinki of the World Medical Association. All women provided informed consent for participation.

### 2.2. Clinical Data and Outcomes

Maternal demographic and clinical data, including age, nationality (Han or other), occupation, education level, smoking history (yes or no), and alcohol consumption history (yes or no), were collected. According to Chinese clinical standards, spontaneous abortion was defined as pregnancy loss before 28 weeks of gestation [1]; the Abortion group was further stratified into two subgroups: the 1-spontaneous abortion group and the ≥2 spontaneous abortions group (RSA). Preterm birth was defined as delivery between 28 and 37 weeks of gestation [13]. PROM was defined as the rupture of fetal membranes prior to the onset of labor contraction [14]. Amniotic fluid characteristics were operationally categorized as clear or meconium-stained (Grades I–III) [15]. Intrapartum maternal fever was defined as a body temperature of 38 °C (100.4 °F) or higher during labor [16].

### 2.3. Vaginal Microecology Test

A sterile cotton swab was used to collect vaginal contents from the upper third of the vaginal wall. One of the collected swabs was utilized to prepare two slide specimens. The first slide was used for microscopic examination of *Trichomonas vaginalis* and to calculate the Donders score using the physiological saline wet mount method. The second slide was employed to assess the density and diversity of vaginal flora, determine the Nugent score, and grade *Lactobacillus*. This slide was examined under oil immersion microscopy after drying, fixing, and Gram staining. Vaginal cleanliness was classified according to the National Clinical Laboratory Practice Guidelines (18th edition): grades I–II were considered normal, while grades III–IV were classified as abnormal. Semi-quantitative assessment of *Lactobacillus* proportion: 10 random high-power oil-immersion fields (100× objective lens) were selected for each smear. All bacterial morphotypes within each field were enumerated, and the ratio of large Gram-positive bacilli consistent with *Lactobacillus* to total bacterial morphotypes was calculated per field. The mean value across the 10 fields was recorded as the *Lactobacillus* proportion for the specimen. The Nugent score was calculated by evaluating the quantities of *Lactobacillus* morphotypes (scored 0–4), *Gardnerella vaginalis* morphotypes (scored 0–4), and *Mobiluncus* morphotypes (scored 0–2). Leukocyte esterase, sialidase, and catalase were detected using the bPR-2014A (V1.7.5) vaginitis automatic analyzer and its detection kit (Master Biotechnology Co., Ltd., Suzhou, Jiangsu, China). Vaginal pH was measured using colorimetric pH strips with a range of 3.8–5.4. Disagreements between two independent examiners were resolved by joint re-review of the slide to achieve consensus.

Diagnostic evaluations were performed based on the Vaginal Microecology Evaluation System (version 2016) [12], as follows: (1) Normal vaginal microecology: density and diversity grades were II–III, the dominant bacteria were *Lactobacillus*, no pathogenic microorganisms were detected, vaginal pH value was 3.8–4.5, *Lactobacillus* grade was I–IIa, and leukocyte esterase and sialidase were negative. (2) BV: Nugent score ≥ 7 points. (3) VVC: fungal spores or pseudohyphae could be found microscopically under oil. (4) AV: according to the clinical manifestations and microscopic Donders score ≥ 3 points. The Donders score is computed from five microscopic components: lactobacillary grade, inflammatory response, proportion of toxic leukocytes, microflora features, and abundance of immature epithelial cells. Scores of 3–4 correspond to mild AV, 5–6 to moderate AV, and 7–10 to severe AV [17]. (5) Trichomoniasis (TV): a large number of white blood cells and active Trichomoniasis under the microscope. (6) Mixed vaginitis: two or more types of vaginitis existing at the same time. All laboratory procedures were conducted according to the manufacturer’s instructions.

### 2.4. Statistical Analyses

SPSS 27.0 was used for statistical analysis. Continuous variables were presented as mean ± standard deviation (SD), while categorical variables were described as *n* (percentage, %) for baseline characteristics. The chi-square (χ^2^) test or Fisher’s exact test was used to compare the distributions of baseline characteristics across different groups, depending on the expected cell frequencies. Multivariable models were adjusted for maternal age, and corresponding odds ratios (ORs) and 95% confidence intervals (CIs) were reported. All statistical tests were two-tailed, and a two-sided *p* value < 0.05 was considered statistically significant.

## 3. Results

### 3.1. Patient Characteristics

Characteristics of 786 participants are described in Table 1. The mean age of the participants was 30.5 ± 4.08 years. 160 cases (20.4%) experienced spontaneous abortion, while 626 cases (79.6%) achieved delivery. With respect to lifestyle-related characteristics, only 14 participants (1.8%) reported a smoking history; 94 participants (12.0%) had a history of alcohol intake. Four subtypes of genital infections were documented in the cohort: 68 cases (8.4%) of BV, 94 cases (12.0%) of VVC, 57 cases (7.3%) of cytolytic vaginosis (CV), and 104 cases (13.2%) of AV, of whom 20 (2.5%) had moderate-to-severe AV. Several pregnancy-related complications were documented and sorted by prevalence: gestational diabetes mellitus (GDM, *n* = 137, 17.4%), PROM (*n* = 131, 16.7%), preterm birth (PTB, *n* = 35, 4.5%), intrapartum fever (*n* = 31, 3.9%), hypertensive disorders of pregnancy (HDP, *n* = 25, 3.2%), and meconium-stained amniotic fluid (*n* = 24, 3.1%).

### 3.2. Association Between Vaginal Microecology and Spontaneous Abortion

To investigate the association between vaginal microecology and the risk of spontaneous abortion, we stratified the entire study cohort into two groups: the spontaneous-abortion group and the non-abortion pregnancy group.

For vaginal microecological parameters, abnormal bacterial density (OR = 3.75, 95% CI = 2.21–6.33, *p* < 0.001), abnormal cleanliness levels (OR = 1.91, 95% CI = 1.33–2.74, *p* < 0.001), and abnormal pH (OR = 1.74, 95% CI = 1.21–2.49, *p* = 0.003) were correlated with abortion risk after adjustment for maternal age (Table 2). The mean proportion of *Lactobacillus* in the abortion group was 42.5 ± 35.85%, which was significantly lower than that in the non-abortion group (61.6 ± 26.72%; *p* < 0.001). Consistently, LBG scores (grade IIb-III, representing *Lactobacillus* depletion) were associated with elevated abortion risk following maternal-age adjustment (OR = 3.44, 95% CI = 2.37–4.99, *p* < 0.001).

Among different types of vaginal infections, VVC (OR = 2.17, 95% CI = 1.34–3.52, *p* = 0.001), BV (OR = 3.19, 95% CI = 1.89–5.38, *p* < 0.001), and moderate-to-severe AV (OR = 2.71, 95% CI = 1.08–6.82, *p* = 0.034) were associated with higher spontaneous-abortion risk following adjustment for maternal age (Table 2). For enzymological indicators, the inflammation-related leukocyte esterase was associated with an elevated abortion risk (OR = 2.22, 95% CI = 1.39–3.61, *p* < 0.001), while the BV-related Sialidase was also linked to an increased abortion risk (OR = 2.68, 95% CI = 1.06–6.80, *p* = 0.037).

### 3.3. Association Between Vaginal Microecology and RSA

We assessed associations between vaginal microecology and RSA among 160 women with abortion outcomes. Notably, VVC was significantly associated with RSA risk after age adjustment (OR = 2.53, 95% CI = 1.11–5.83, *p* = 0.028). No significant associations with RSA were observed for abnormal BV, AV, catalase, sialidase, and leukocyte esterase following age adjustment (Table 3). Future studies with larger cohorts are warranted to clarify these potential relationships.

### 3.4. Association Between BV and Pregnancy Outcomes

To examine the association between BV and birth outcomes, we analyzed the links between BV and indicators including preterm birth, PROM and so on (Table 4). Results showed that BV was significantly associated with an elevated risk of PROM (OR = 2.95, 95% CI = 1.50–5.79, *p* = 0.002). No significant associations were observed between BV and preterm birth, meconium-stained amniotic fluid, or intrapartum fever.

## 4. Discussion

Among the multiple etiologies of spontaneous abortion, the role of the vaginal microbiota is often overlooked. In fact, the oral microbiome, gastrointestinal microbiome, and urogenital microbiome may modulate pregnancy-related outcomes [18]. Nevertheless, the present study focuses primarily on the vaginal microbiome. Vaginal microbiota contributes to maintaining the balance of vaginal acidity, pH, and metabolic profiles, thereby helping to prevent spontaneous abortion [19]. In the present study, we examined the impact of first-trimester vaginal microecology on pregnancy outcomes; our findings revealed that the proportion of *Lactobacillus* in the vaginal microenvironment of women with spontaneous abortion was significantly reduced. Vaginal inflammatory conditions, particularly VVC and BV, were identified as independent risk factors for spontaneous abortion. Additionally, inflammation-related markers (leukocyte esterase) were also associated with an elevated risk of spontaneous abortion.

*Lactobacillus* is the dominant genus in the vagina and cervix in healthy women, and its reduced abundance may cause an infection state and be related to infertility. A large-scale Chinese cohort study from Peking Union Medical College Hospital demonstrated greater perturbation of vaginal microbial networks among women who subsequently experienced preterm birth relative to term-birth controls, accompanied by marked depletion of *Lactobacillus* [20]. Our study found that the mean proportion of *Lactobacillus* in the abortion group was 42.5 ± 35.85%, which was significantly lower than that in the non-abortion group (61.6 ± 26.72%; *p* < 0.001). Consistent with this observation, prior research has documented that 77.3% of spontaneous abortion cases correspond to vaginal microbiome community-state types (CSTs) that are not dominated by *Lactobacillus* [21].

With advances in microbial technologies, vaginal *Lactobacillus* can be classified into several distinct species, including *Lactobacillus crispatus, L. jensenii, L. gasseri, and L. iners* [22]. *L. crispatus* exerts protective effects against pathogenic colonization: it achieves this via competitive exclusion, impairment of pathogen-epithelial cell adhesion, and secretion of antimicrobial molecules (e.g., bacteriocins, lactic acid) as well as immunomodulatory factors. Vaginal microbiota dominated by *L. iners* exhibit poor stability and are prone to transitioning toward non-*Lactobacillus*-dominated profiles among women with PTB. This shift represents a key mechanism underlying PTB [23]. Several clinical trials have evaluated the clinical benefits of *Lactobacillus*-containing preparations for pregnancy outcomes (NCT05156333; CRD42023421613). Following oral probiotic capsule intervention administered from gestational week 30 to 37, no significant improvements in clinical pregnancy outcomes were observed. Nevertheless, maternal probiotic supplementation effectively modulated breast-milk and infant gut microbiota and markedly reduced the relative abundance of *Gardnerella vaginalis*, driving favorable shifts [24,25,26]. Further studies are required to clarify whether these microbial shifts can translate into tangible clinical benefits.

Grewal et al. validated this immunomodulatory function by assessing cervicovaginal contents and vaginal microbiota compositions: relative to women with term births, individuals experiencing abortion (who also had a *Lactobacillus*-depleted vaginal microbiota) exhibited higher concentrations of proinflammatory cytokines (interleukin-1β(IL-1β), interleukin-6(IL-6), and tumor necrosis factor-α (TNF-α) [27]. Notably, *L. iners* provides less robust protection against cervicovaginal infections than other vaginal *Lactobacillus* species. For example, D-lactic acid-a key antimicrobial metabolite—is synthesized by *L. crispatus, L. gasseri, and L. jensenii*, but is not produced by *L. iners* [28].

This study also found that BV and the sialidase it secretes serve as risk factors for spontaneous abortion. BV can be diagnosed via multiple approaches, with the Nugent score being the most commonly utilized. Scores are defined as 0–3 for a *Lactobacillus*-dominated vaginal microbiota and 7–10 for BV, characterized by reduced *Lactobacillus* abundance and increased levels of Nugent-BV-associated taxa. BV is typified by depletion of beneficial *Lactobacillus* species and overgrowth of diverse anaerobic vaginal taxa, including *Gardnerella, Prevotella,* and *Mobiluncus* spp. [29]. Notably, *Atopobium*, *Prevotella*, and *Gardnerella* constitute key pathogenic taxa in BV, a condition that shows a significant association with spontaneous abortion (OR = 1.680, 95% CI: 1.146–2.462; *p* = 0.011) [30]. Women who develop preterm birth display markedly higher species-level genomic nucleotide diversity within their vaginal microbiota, especially during the first trimester. Such elevated genetic diversity is primarily driven by the genus *Gardnerella*, which comprises multiple distinct species and putative novel taxa including *G. piotti*, *G. swidsinskii*, and *G. gs13* [20,31]. *Gardnerella vaginalis* exerts pathogenic effects via multiple mechanisms, including adhering to vaginal epithelial cells, promoting sialidase and vaginolysin secretion, inducing the release of proinflammatory cytokines, and facilitating biofilm formation [32,33].

Our findings indicated that BV may also contribute to adverse pregnancy outcomes such as PROM, whereas no significant associations were observed between BV and meconium-stained amniotic fluid or intrapartum fever. Conflicting evidence exists regarding the link between BV and PTB. A previous study reported that Nugent scores of 7–10 (diagnostic of BV) in early pregnancy (OR = 0.84) and late pregnancy (aRR: 1.04) were not associated with PTB [34]. However, other research has demonstrated that BV was significantly associated with PTB (OR = 1.76), low birth weight (OR = 1.73), and birth asphyxia (OR = 2.90). Among maternal outcomes, PROM exhibited the highest BV prevalence (13.2%, 95% CI 6.1–22.3%) [35]. This discrepancy may be attributed to the insufficient sample size of BV-positive cases in the present study, which could have introduced bias.

Collectively, vaginal microecology constitutes an integrated biological system. Investigators from Virginia Commonwealth University analyzed metagenomic sequencing data of 705 vaginal samples from the iHMP-supported Multi-Omic Microbiome Study-Pregnancy Initiative (MOMS-PI), including 40 cases of PTB and 135 term-birth controls. This study first identified *L. crispatus* as a protective taxon, while BVAB1, *Sneathia amnii*, TM7-H1, and *Prevotella* cluster 2 constituted key high-risk taxa for PTB, and developed a first-trimester four-taxon predictive model with an accuracy of 72.3% [31]. Mechanistically, *Gardnerella vaginalis, Prevotella, and Sneathia* spp. generate substantial amounts of reactive oxygen species (ROS) during their metabolic processes. Excessive ROS production induces oxidative damage to fetal membranes. Furthermore, oxidative stress impairs signaling pathways in fetal membranes, particularly via the activation of nuclear factor-κB (NF-κB) and mitogen-activated protein kinase (MAPK) pathways. Such activation enhances the production of proinflammatory mediators, which in turn amplifies the inflammatory response and promotes the activity of matrix metalloproteinases (MMPs) [36]. This forms a vicious cycle wherein the synergistic effects of oxidative stress and inflammation drive the rapid degradation of fetal membranes, ultimately leading to PROM [37,38]. In addition, bacterial capsular polysaccharide (CPS), an acidic lipopolysaccharide-related molecule, impairs antigen-presenting function of immature dendritic cells, disturbs T-cell activation and natural-killer-cell migration, and facilitates bacterial evasion of host immune defenses [39]. Tryptophan metabolites (indoles) derived from the microbiota activate the aryl hydrocarbon receptor (AhR) signaling pathway, which induces myeloid-derived suppressor cells (MDSCs) and gut-derived RORγt^+^ regulatory T cells (Tregs). These events further suppress IFN-γ^+^ T-cell and IL-17A^+^ T-cell responses at the maternal-fetal interface [40].

The present study has several limitations. Firstly, this single-center hospital-based cohort may not fully represent community-dwelling low-risk pregnant women, as women with abortion-related signs were more willing to receive testing. Serial sampling across trimesters would improve accuracy given dynamic vaginal microecology. Secondly, vaginal microecology was evaluated using conventional wet-mount microscopy for clinical practicability (rapid one-hour turnaround for patient care). Nevertheless, 16S rRNA or metagenomic sequencing would offer more detailed information on microbial composition and functional pathways. We plan to adopt these sequencing approaches in future work.. Emerging machine-learning approaches leveraging large-scale gut metagenomic datasets enable identification of previously uncharacterized microbial genomes and proteins [41]. Thirdly, Key covariates, including vaginitis-associated symptoms, clinical signs, chronic diseases, medication use, and exposure to other pathogens, were not adequately documented. Regression models were adjusted solely for maternal age; residual confounding from gestational age at clinical interventions and concurrent vaginal infections cannot be ruled out. More complete recording of clinical manifestations should be emphasized in future investigations.

## 5. Conclusions

In summary, this study demonstrated lower *Lactobacillus* abundance in the vaginal microenvironment among women experiencing spontaneous abortion, and identified BV and VVC as independent risk factors for spontaneous abortion. Furthermore, BV was associated with PROM. These findings underscore the need for early vaginal microecological monitoring. Further prospective studies are warranted to explore whether interventions targeting vaginal dysbiosis (e.g., vaginal *Lactobacillus* preparations) may help mitigate adverse pregnancy-related outcomes.

## Figures and Tables

**Table 1:** Characteristics of participants.

Patient Characteristics	Case Count (N = 786)	Percent (%)
Age, years	30.5 ± 4.08	
Pregnancy Outcome		
Abortion	160	20.4%
Non-abortion pregnancy group	626	79.6%
Ethnicity		
Han ethnicity	762	96.9%
Ethnic minorities	24	3.1%
Occupational status		
Employed	676	86.0%
Unemployed	110	14.0%
Smoking history		
Previous smoker	14	1.8%
Never smoker	772	98.2%
Alcohol drinking history		
Previous drinkers	94	12.0%
Never drinkers	692	88.0%
Genital infections		
BV	68	8.4%
VVC	94	12.0%
CV	57	7.3
AV	104	13.2%
Complications		
PROM	131	16.7%
PTB	35	4.5%
Intrapartum Fever	31	3.9%
meconium-stained amniotic fluid	24	3.1%
HDP	25	3.2%
GDM	137	17.4%

Abbreviations: BV, bacterial vaginosis; VVC, vulvovaginal candidiasis; CV, cytolytic vaginosis; AV, aerobic vaginitis; PROM, premature rupture of membranes; PTB, preterm birth; GDM, gestational diabetes mellitus; and HDP, hypertensive disorders of pregnancy.

**Table 2:** Comparison of vaginal microenvironment changes in abortion and non-abortion women.

Characteristics, *n* (%)	Abortion (*n* = 160)	Non-Abortion (*n* = 626)	Unadjusted		Age Adjusted	
			OR (95% CI)	*p*	OR (95% CI)	*p*
Bacterial Density						
Normal (*n* = 720)	130 (81.2%)	590 (94.2%)	Ref.			
Abnormal (*n* = 66)	30 (18.8%)	36 (5.8%)	3.78 (2.24–6.36)	<0.001	3.75 (2.21–6.33)	<0.001
Bacterial Diversity						
Normal (*n* = 773)	157 (98.1%)	616 (98.4%)	Ref.			
Abnormal (*n* = 13)	3 (1.9%)	10 (1.6%)	0.85 (0.23–3.12)	0.806	1.24 (0.34–4.59)	0.745
Cleanliness Levels						
Normal (*n* = 391)	59 (36.9%)	332 (53.0%)	Ref.			
Abnormal (*n* = 395)	101 (63.1%)	294 (47.0%)	1.93 (1.35–2.76)	<0.001	1.91 (1.33–2.74)	<0.001
PH						
Normal (*n* = 382)	59 (36.9%)	323 (51.6%)	Ref.			
Abnormal (*n* = 404)	101 (63.1%)	303 (48.4%)	1.82 (1.27–2.61)	<0.001	1.74 (1.21–2.49)	0.003
LBG						
Normal (*n* = 589)	86 (53.8%)	503 (80.4%)	Ref.			
Abnormal (*n* = 197)	74 (46.3%)	123 (19.6%)	3.52 (2.43–5.09)	< 0.001	3.44 (2.37–4.99)	< 0.001
Fungal hypha						
Negative (*n* = 734)	137 (85.6%)	597 (95.4%)	Ref.			
Positive (*n* = 52)	23 (14.4%)	29 (4.6%)	3.46 (1.93–6.16)	<0.001	3.78 (2.10–6.81)	<0.001
Fungal spore						
Negative (*n* = 692)	130 (81.3%)	562 (89.8%)	Ref.			
Positive (*n* = 94)	30 (18.8%)	64 (10.2%)	2.03 (1.26–3.25)	0.003	2.17 (1.34–3.52)	0.001
VVC						
Negative (*n* = 692)	130 (81.3%)	562 (89.8%)	Ref.			
Positive (*n* = 94)	30 (18.8%)	64 (10.2%)	2.03 (1.26–3.25)	0.003	2.17 (1.34–3.52)	0.001
BV						
Negative (*n* = 718)	131 (81.9%)	587 (93.8%)	Ref.			
Positive (*n* = 68)	29 (18.1%)	39 (6.2%)	3.33 (1.98–5.58)	<0.001	3.19 (1.89–5.38)	<0.001
Nugent Score						
0–3 (*n* = 623)	94 (58.8%)	529 (84.5%)	Ref.			
4–6 (*n* = 95)	37 (23.1%)	58 (9.3%)	3.59 (2.12–6.07)	<0.001	3.48 (2.17–5.57)	<0.001
≥7 (*n* = 68)	29 (18.1%)	39 (6.2%)	4.18 (2.47–7.10)	<0.001	4.01 (2.35–6.82)	<0.001
AV						
Normal or mild (*n* = 766)	152 (95.0%)	614 (98.1%)	Ref.			
Moderate-to-severe (*n* = 20)	8 (5.0%)	12 (1.9%)	2.69 (1.08–6.70)	0.033	2.71 (1.08–6.82)	0.034
Catalase						
Negative (*n* = 170)	32 (20.0%)	138 (22.0%)	Ref.			
Positive (*n* = 616)	128 (80.0%)	488 (78.0%)	1.16 (0.75–1.81)	0.485	1.08 (0.70–1.67)	0.718
Sialidase						
Negative (*n* = 688)	132 (82.5%)	556 (88.8%)	Ref.			
Positive (*n* = 98)	28 (17.5%)	70 (11.2%)	2.65 (1.05–6.68)	0.038	2.68 (1.06–6.80)	0.037
Leukocyte esterase						
Negative (*n* = 351)	38 (23.8%)	313 (50.0%)	Ref.			
Positive (*n* = 435)	122 (76.3%)	313 (50.0%)	2.19 (1.36–3.51)	0.001	2.22 (1.39–3.61)	<0.001

Notes: Abbreviations: BV, bacterial vaginosis; VVC, vulvovaginal candidiasis; CV, cytolytic vaginosis; AV, aerobic vaginitis; LBG, *Lactobacillus* grade.

**Table 3:** Comparison of vaginal microenvironment changes in RSA women.

Characteristics, *n* (%)	Non-RSA (*n* = 117)	RSA (*n* = 43)	Unadjusted		Age Adjusted	
			OR (95% CI)	*p*	OR (95% CI)	*p*
Bacterial Density						
Normal (*n* = 130)	94 (80.3%)	36 (83.7%)	Ref.			
Abnormal (*n* = 30)	23 (19.7%)	7 (16.3%)	0.79 (0.31–2.01)	0.628	1.25 (0.49–3.17)	0.632
Cleanliness Levels						
Normal (*n* = 59)	40 (34.2%)	19 (44.2%)	Ref.			
Abnormal (*n* = 101)	77 (65.8%)	24 (55.8%)	1.52 (0.74–3.10)	0.247	1.51 (0.73–3.11)	0.260
PH						
Normal (*n* = 59)	42 (35.9%)	17 (39.5%)	Ref.			
Abnormal (*n* = 101)	75 (64.1%)	26 (60.5%)	1.16 (0.56–2.39)	0.67	1.15 (0.55–2.38)	0.70
Fungal hypha						
Negative (*n* = 137)	101 (86.3%)	36 (83.7%)	Ref.			
Positive (*n* = 23)	16 (13.7%)	7 (16.3%)	0.81 (0.32–2.02)	0.658	0.82 (0.31–2.18)	0.698
Fungal spore						
Negative (*n* = 130)	100 (85.5%)	30 (69.8%)	Ref.			
Positive (*n* = 30)	17 (14.5%)	13 (30.2%)	2.47 (1.10–5.56)	0.028	2.53 (1.11–5.83)	0.028
VVC						
Negative (*n* = 130)	100 (85.5%)	30 (69.8%)	Ref.			
Positive (*n* = 30)	17 (14.5%)	13 (30.2%)	2.47 (1.10–5.56)	0.028	2.53 (1.11–5.83)	0.028
BV						
Negative (*n* = 131)	99 (84.6%)	31 (72.1%)	Ref.			
Positive (*n* = 29)	18 (15.4%)	12 (27.9%)	2.12 (0.92–4.90)	0.076	2.16 (0.93–5.01)	0.071
LBG						
Normal (*n* = 86)	60 (51.3%)	26 (60.5%)	Ref.			
Abnormal (*n* = 74)	57 (48.7%)	17 (39.5%)	0.68 (0.33–1.41)	0.303	0.69 (0.33–1.42)	0.319
AV						
Negative (*n* = 135)	95 (81.2%)	40 (93.0%)	Ref.			
Positive (*n* = 25)	22 (18.8%)	3 (7.0%)	0.32 (0.09–1.14)	0.080	1.28 (0.79–2.10)	0.078
Catalase						
Negative (*n* = 32)	27 (23.1%)	5 (11.6%)	Ref.			
Positive (*n* = 128)	90 (76.9%)	38 (88.4%)	0.44 (0.16–1.22)	0.116	0.44 (0.15–1.22)	0.117
Sialidase						
Negative (*n* = 132)	100 (85.5%)	32 (74.4%)	Ref.			
Positive (*n* = 28)	17 (14.5%)	11 (25.6%)	0.49 (0.20–1.18)	0.107	0.48 (0.20–1.15)	0.100
Leukocyte Esterase						
Negative (*n* = 38)	26 (22.2%)	12 (27.9%)	Ref.			
Positive (*n* = 122)	91 (77.8%)	31 (72.1%)	1.36 (0.60–3.07)	0.457	1.34 (0.60–2.99)	0.474

**Table 4:** Association between BV and pregnancy outcomes in abortion and non-abortion pregnancy women.

Characteristics, *n* (%)	BV Negative (*n* = 587)	BV Positive (*n* = 39)	Unadjusted		Age Adjusted	
			OR (95% CI)	*p*	OR (95% CI)	*p*
Gestational age						
Term (*n* = 591)	553 (94.2%)	38 (97.4%)	Ref.			
Preterm (*n* = 35)	34 (5.8%)	1 (2.6%)	0.43 (0.06–3.21)	0.409	0.43 (0.05–3.24)	0.414
PROM						
No (*n* = 495)	472 (80.4%)	23 (59.0%)	Ref.			
Yes (*n* = 131)	115 (19.6%)	16 (41.0%)	2.85 (1.46–5.57)	0.002	2.95 (1.50–5.79)	0.002
Amniotic Fluid						
Clear (*n* = 602)	565 (96.3%)	37 (94.9%)	Ref.			
Meconium-stained fluid (*n* = 24)	22 (3.7%)	2 (5.1%)	0.72 (0.16–3.18)	0.665	0.75 (0.17–3.33)	0.71
Intrapartum Fever						
No (*n* = 595)	559 (95.2%)	36 (92.3%)	Ref.			
Yes (*n* = 31)	28 (4.8%)	3 (7.7%)	0.60 (0.17–2.07)	0.420	0.56 (0.16–1.95)	0.365
GDM						
No (*n* = 489)	460 (78.4%)	29 (74.4%)	Ref.			
Yes (*n* = 137)	127 (21.6%)	10 (25.6%)	0.80 (0.38–1.69)	0.569	1.05 (0.97–1.14)	0.655
HDP						
No (*n* = 601)	564 (96.1%)	37 (94.9%)	Ref.			
Yes (*n* = 25)	23 (3.9%)	2 (5.1%)	0.76 (0.17–3.32)	0.710	0.74 (0.16–3.27)	0.692

Abbreviations: PROM, premature rupture of membranes; BV, bacterial vaginosis; GDM, gestational diabetes mellitus; HDP, hypertensive disorders of pregnancy.

## Data Availability

The original contributions presented in this study are included in the article. Further inquiries can be directed to the corresponding author(s).
